# A Case of Pseudo-Hermaphrodism

**Published:** 1898-11

**Authors:** R. Sorel, M. Cherot


### A CASE OF PSEUDO-HERMAPHRODISM.

BY DRS. R. SOREL AND M. CHEROT.

Although similar cases are not absolutely rare, the authors deem the report of the above case sufficiently interesting for publication because of the opportunity of following it to the autopsy.

A. C., bar-maid, residing at Havre, was a vigorous-looking individual, muscular, of medium height, and about 36 years old. She had received a girl's education and had always passed as a member of the female sex. She had never menstruated. Her figure was rather masculine in shape. Since the age of 21 a black growth of hair about the lips and chin, although rather scanty, had necessitated resort to shaving. The mammary glands were undeveloped. A considerable hairy growth on the lower limbs was present. General aspect, gait, and voice were those of a man.

In the place of the clitoris this person had a projection emerging at the point of separation of the labia majora. This terminated in an imperforate glans penis of normal size and shape. The projecting fleshy mass was 5½ cm. long and measured 6 cm. in circumference at the root. Beneath it a short band prevented complete erection. This band was lost in two large labia, which were well formed and covered with hair. Between them and beneath the roots of the projection the urethra was visible. In the labia majora no sign of testicles were discoverable, and between them no sign of vulvar or vaginal orifice could be made out. * * * Although of a frigid disposition this person had erections with a desire, which however was never satisfied, of attempting intercourse with women.

Two years previously she received a blow in the right side of the abdomen which caused a severe pain for a number of days. At the time of writing she was sick six weeks with a pain in the right abdominal region. With this she complained of loss of appetite, vomiting and slight diarrhea. * * * On admission to the hospital an abdominal tumor was discovered extending from the right loin to the median line and from three finger-breadths below the liver to the iliac fossa.

On opening the abdomen a half-litre of brown feculent pus escaped. With the finger in the wound cancer of caecum was diagnosticated. On the following day the dressing was soiled with abundant fecal matter and the patient died early the next morning.

The autopsy showed invasion of the right side of the abdomen by a vast cancerous growth, purulent collections, and generally peritonitis. The search for testicles, uterus, tubes, ovaries and ligaments proved futile. The bladder was relatively small. Behind the bladder a pocket, 8 cm. x 6 cm., was found lined with mucous membrane and filled with liquid. At its lower portion it communicated with the urethra. On the the peritoneal surface below to either side the ureters were discovered. Testicles could be found nowhere. The urethra w s short like that of a female. No prostate gland present. The seminal vesicles were found.—*The Post-Graduate.*

